# Randomized Clinical Trial of Antibiotic Therapy for Antenatal Pyelonephritis

**DOI:** 10.1155/S1064744996000567

**Published:** 1996

**Authors:** Brian C. Brost, Barry Campbell, Sue Stramm, Daniel Eller, Roger B. Newman

**Affiliations:** Division of Maternal-Fetal Medicine Department of Obstetrics and Gynecology Medical University of South Carolina Charleston South Carolina 29425 USA

## Abstract

*Objective:* The aim of this study was to prospectively evaluate the efficacy of a therapeutic course of intravenous antibiotics followed by oral antibiotics vs. intravenous antibiotics alone to prevent recurrent urinary tract infection.

*Methods:* Pyelonephritis was documented by strict criteria in 67 pregnant women who were then treated with appropriate intravenous antibiotics until afebrile and asymptomatic for 48 h. Women were then randomized to completion of a 10-day course of oral nitrofurantoin 100 mg qid or no further antibiotic therapy. Urine cultures (UC) were obtained 2 and 6 weeks after discharge, and at delivery. A positive UC or readmission for pyelonephritis was considered endpoints for participation in the study. Antibiotic suppression was not used.

*Results:* Readmission for pyelonephritis prior to the 2-week follow-up visit occurred in 2/36 (5.6%) women randomized to the oral therapy group compared to 4/31 (12.9%) in the no oral treatment group (*P* = 0.29). At the 2-week follow-up visit, 6/31 (19.4%) women had a positive UC in the oral therapy compared to 8/26 (30.1%) in the no oral treatment group but this difference was not statistically significant (*P* = 0.31).

*Conclusions:* Completion of 10 days of antibiotic therapy with oral medication does not significantly reduce the risk of recurrent pyelonephritis immediately post-parenteral treatment. Women in the no oral treatment group had a non-significant increase in positive UC at the 2-week follow-up visit. The high rates of recurrent urinary tract infection during pregnancy in both groups underscore the need for regular UC and for the possible role of oral antibiotic suppression.


Infectious Diseases in Obstetrics and Gynecology 4:294–297 (1996)
© 1997 Wiley-Liss, Inc.

### Randomized Clinical Trial of Antibiotic Therapy for Antenatal Pyelonephritis

**Brian C. Brost,* Barry Campbell, Sue Stramm, Daniel Eller, and Roger B. Newman**

*Division of Maternal-Fetal Medicine, Department of Obstetrics and Gynecology, Medical University of South Carolina, Charleston, South Carolina*

### Abstract

**Objective:** The aim of this study was to prospectively evaluate the efficacy of a therapeutic course of intravenous antibiotics followed by oral antibiotics vs. intravenous antibiotics alone to prevent recurrent urinary tract infection.

**Methods:** Pyelonephritis was documented by strict criteria in 67 pregnant women who were then treated with appropriate intravenous antibiotics until afebrile and asymptomatic for 48 h. Women were then randomized to completion of a 10-day course of oral nitrofurantoin 100 mg qid or no further antibiotic therapy. Urine cultures (UC) were obtained 2 and 6 weeks after discharge, and at delivery. A positive UC or readmission for pyelonephritis was considered endpoints for participation in the study. Antibiotic suppression was not used.

**Results:** Readmission for pyelonephritis prior to the 2-week follow-up visit occurred in 2/36 (5.6%) women randomized to the oral therapy group compared to 4/31 (12.9%) in the no oral treatment group ($P = 0.29$). At the 2-week follow-up visit, 6/31 (19.4%) women had a positive UC in the oral therapy compared to 8/26 (30.1%) in the no oral treatment group but this difference was not statistically significant ($P = 0.31$).

**Conclusions:** Completion of 10 days of antibiotic therapy with oral medication does not significantly reduce the risk of recurrent pyelonephritis immediately post-parenteral treatment. Women in the no oral treatment group had a non-significant increase in positive UC at the 2-week follow-up visit. The high rates of recurrent urinary tract infection during pregnancy in both groups underscore the need for regular UC and for the possible role of oral antibiotic suppression. Infect. Dis. Obstet. Gynecol. 4:294–297, 1996. © 1997 Wiley-Liss, Inc.

Key words pregnancy; urinary infection; asymptomatic bacteriuria; antenatal pyelonephritis

Pyelonephritis during pregnancy is a serious complication that often results in significant maternal and neonatal morbidity.[1] The treatment of antenatal pyelonephritis typically includes a course of parenteral antibiotics until the patient becomes asymptomatic followed by oral antibiotics to complete a 10–14-day antibiotic regimen. Completion of oral therapy depends on the compliance of the patient. If women with antenatal pyelonephritis could be managed as effectively with only parenteral antibiotics until afebrile and asymptomatic for 48 h, this would result not only in a potential cost savings, but possibly would also circumvent the compliance issue entirely.

We propose to evaluate the effectiveness of parenteral therapy alone against the more traditional regimen which includes a follow-up course of oral antibiotics to prevent recurrent pyelonephritis and

*Correspondence to: Dr. Brian C. Brost, Department of Obstetrics and Gynecology, 171 Ashley Avenue, Charleston, SC 29425.

Received 15 August 1996
Accepted 14 January 1997

asymptomatic urinary tract infections. Close surveillance of urine cultures after completion of antibiotic therapy was chosen instead of using antibiotic suppression as was suggested by Lenke et al.[2] This management protocol allows us to more adequately evaluate the early and late infectious sequelae of the proposed antibiotic regimens.

### SUBJECTS AND METHODS

During the study period (August 1990 to December 1994), women admitted for antepartum pyelonephritis at the Medical University of South Carolina were screened for protocol eligibility. Study entry requirements were based on a strict set of screening diagnostic criteria including an admission oral temperature ⩾38.0°C, costovertebral angle tenderness, and a positive urine culture (UC) with ⩾$10^5$ colony forming units (CFU) of a pathogenic urinary organism obtained prior to initiation of antibiotic therapy. Exclusion criteria included evidence of a renal abscess, a prior episode of pyelonephritis during the index pregnancy, and women not exhibiting *all* of the inclusion criteria.

The initial antibiotic regimen was determined by the attending physician. Antibiotic therapy was continued until the patient was asymptomatic on physical examination and was afebrile (temperature <38.0°C) for at least 48 h. Patients were approached prior to completion of intravenous antibiotic therapy about participation in our study protocol and randomized after obtaining written consent. Repeat UCs were not obtained prior to discharge from the hospital. The study protocol was approved by the Institutional Review Board at our facility.

Patients entering the protocol were randomized into one of two treatment groups by a coin-flip table established at the beginning of the study. Women in the first group received a total of 10 days of antibiotic therapy (intravenous followed by oral nitrofurantoin 100 mg qid), while the other group of women received no further antibiotic therapy after completion of intravenous therapy. Women enrolled in the study were followed for evidence of reinfection until their delivery. Clean catch UCs were obtained at 2 and 6 weeks after discharge from the hospital and again at delivery. Recurrent pyelonephritis or a positive UC after documented cure was considered signs of reinfection. No suppressive antibiotic therapy was used in any of the study patients. Women were removed from the study at the time of any positive UC or episode of recurrent pyelonephritis. At that point, they were retreated with appropriate antibiotic therapy and placed on antibiotic suppression.

The data obtained were analyzed using a two-tailed Student's *t*-test for comparison of means and chi-square analysis or Fisher's exact test as appropriate for evaluation of categorical variables. Values were considered statistically significant at $P < 0.05$. Power calculations performed after the data were collected and before the data were analyzed showed that 31 women would be needed in each group to show a 35% difference in recurrent urinary tract infections at an alpha of 0.05 and a beta of 0.2.

### RESULTS

During the study period, all pregnant women admitted with the diagnosis of antenatal pyelonephritis were screened for study participation. All 67 women meeting the entry criteria agreed to participate. The oral therapy group was comprised of 36 women, and 31 were randomized to receive no further oral antibiotic therapy. The admission demographics (mean ± SD) including maternal age, ethnic composition, estimated gestational age on admission, maternal temperature on admission, white blood cell count on admission, urinary pathogen, antibiotic therapy, and days on intravenous antibiotics were similar between the two groups (Table 1).

During the 2-week interval after discharge from the hospital, readmission for acute pyelonephritis occurred in 2/36 (5.6%) of the women randomized to oral therapy and 4/31 (12.9%) of the women in the no oral treatment group ($P = 0.29$). Delivery also occurred in 4 patients prior to the 2-week culture. In these 4 women there was a positive UC in 1, a negative UC in 2, and no culture obtained in 1. At the 2-week evaluation, a positive UC was noted in 6/31 (19.4%) and 8/26 (30.1%) of the women in the oral therapy and no oral treatment groups, respectively ($P = 0.31$). Combining the positive UC results to the readmissions for pyelonephritis, a total of 9/36 (25.0%) women in the oral therapy group and 12/31 (38.7%) women in the no oral therapy group had evidence of reinfection within 2 weeks of initial intravenous therapy for antenatal pyelonephritis ($P = 0.22$).

During the remainder of the pregnancy, pyelo-

**TABLE 1. Maternal admission characteristics and hospital course[a]**

| | Oral antibiotics (n = 36) | No further therapy (n = 31) | |
|---|---|---|---|
| Age (years) | 22.7 ± 5.04 | 21.5 ± 4.11 | NS |
| Race | | | |
| White | 28 (77.8%) | 17 (54.8%) | NS |
| Black | 7 (19.4%) | 12 (38.7%) | NS |
| Other | 1 (2.8%) | 2 (6.5%) | NS |
| EGA at admission (weeks) | 21.9 ± 7.75 | 24.9 ± 6.76 | NS |
| Range (weeks) | (9.0–38.1) | (10.7–38.6) | |
| Temperature on admission (°C) | 38.9 ± 0.56 | 39.1 ± 0.62 | NS |
| WBC on admission | 13.4 ± 3.96 | 14.2 ± 4.14 | NS |
| Organism | | | |
| *Escherichia coli* | 32 (88.9%) | 26 (83.9%) | NS |
| *Proteus* | 0 | 1 (3.2%) | NS |
| *Klebsiella* | 3 (8.3%) | 1 (3.2%) | NS |
| *Enterobacter* | 0 | 1 (3.2%) | NS |
| Other | 1 (2.8%) | 2 (6.5%) | NS |
| Antiobiotic therapy | | | |
| Cefazolin | 26 (72.2%) | 22 (71.0%) | NS |
| Cefazolin/gentamicin | 7 (19.4%) | 5 (16.1%) | NS |
| Cefazolin/other | 2 (5.6%) | 2 (6.5%) | NS |
| Ampicillin/gentamicin | 1 (2.8%) | 1 (3.2%) | NS |
| Other | 0 | 1 (3.2%) | NS |
| Days IV antibiotics | 4.5 ± 2.46 | 4.6 ± 1.69 | NS |
| EGA at delivery (weeks) | 38.2 ± 3.45 | 38.8 ± 2.50 | NS |
| Range (weeks) | (17.1–41.4) | (34.3–41.3) | |

[a]NS, not statistically significant; EGA, estimated gestational age; WBC, white blood cell count; IV, intravenous.

nephritis recurred in 2 more women in the oral therapy group and 1 in the no oral treatment group. Positive UCs following the initial 2-week check were noted in 6/36 (16.7%) women in the oral therapy group and 2/31 (6.5%) in the no oral treatment group. The organisms grown on UC from the initial admission for pyelonephritis and each subsequent episode of urinary tract infection were similar in all cases obtained (9/12). On 3 of the recurrent admissions, the UC failed to grow an organism or was not obtained. These women exhibited all the classical signs and symptoms of pyelonephritis and were treated as infected.

### DISCUSSION

In this study, it appears that the combination of parenteral antibiotics followed by a 10-day course of oral antibiotics for 10 days does not significantly reduce the short-term risk of recurrent pyelonephritis and urinary tract infection. Long term, the total number of episodes of recurrent infection after admission for pyelonephritis was similar in both groups through delivery (47.2% vs. 48.4% in the oral therapy and no oral therapy groups, respectively). However, with review of the entire study group throughout pregnancy, two trends become apparent (Fig. 1). The majority of the cases of pyelonephritis (4/5) and positive UCs (8/10) in women receiving parenteral therapy only occurs within 2 weeks of discharge. In contrast, the group receiving a 10-day course of oral antibiotics had a higher rate of recurrent infection after the initial 2-week culture check.

These findings lead one to consider two important points: 1) the treatment of pyelonephritis during pregnancy may require more aggressive therapy (a longer intravenous antibiotic course than until asymptomatic and afebrile for 48 h) given the number of positive cultures and readmissions for pyelonephritis noted in both groups, and 2) the importance of close surveillance for recurrent or persistent urinary tract infection as suggested by Lenke et al.[2]

One other study by Faro et al.[3] addresses the issue of treating antenatal pyelonephritis with parenteral antibiotics alone. Women were treated with cefuroxime for a minimum of 5 days and until they were clinically asymptomatic and afebrile for more than 72 h. Of the 23 patients enrolled in the study with antenatal pyelonephritis, 12 (52%) were considered both a clinical and bacteriologic cure by negative culture results at 2–3 weeks after discharge. The other women were classified either as colonized (6/23) or lost to follow-up (5/23). Their rates of recurrent or "colonized" urinary tract infections were similar to the findings of women not treated with oral antibiotics in our study.

The patient return rate in our study is typical of a lower socioeconomic clinic population with about 1/3 of the women enrolled in the study not returning for their 2-week culture check (9 women in the no oral therapy group and 8 women in the oral therapy group). While these numbers seem elevated, only 5 women had no recurrent urinary tract disease or UC obtained through the remainder of their pregnancies (3 in the no oral therapy group and 2 in the oral therapy group). This fact would also call into question the compliance of women in the oral antibiotic treatment group. It is possible

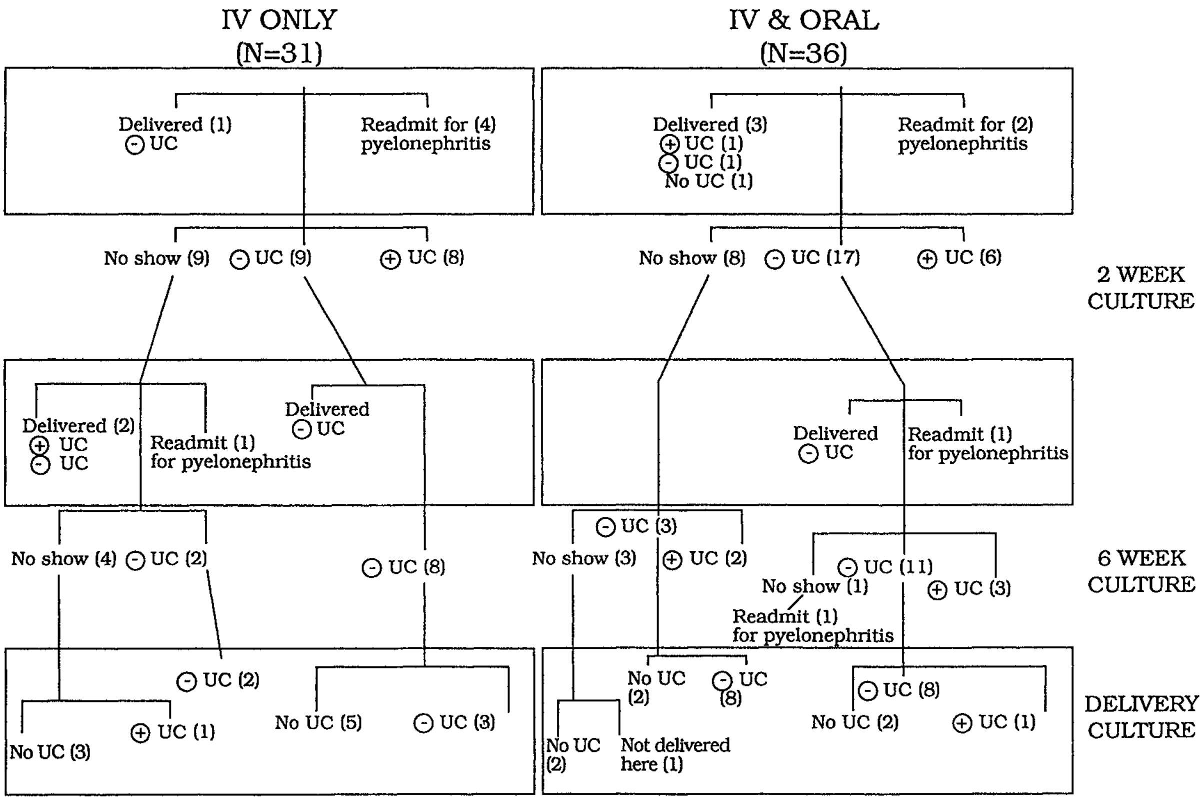

Fig. 1. UC results and timing of recurrent urinary tract infection.

that some of the early recurrent infections in the oral therapy group could represent women who were non-compliant in completing their course of nitrofurantoin.

While the use of parenteral antibiotics was successful in treating antenatal pyelonephritis, those women not started on a course of subsequent oral antibiotics showed a trend toward early recurrence of urinary tract infection. However, a course of intravenous and oral antibiotic therapy does not significantly reduce the risk of recurrent pyelonephritis in the 2 weeks following discharge from the hospital. The high rates of recurrent urinary tract infection during pregnancy in both groups underscore the need for regular follow-up with UC or possible oral antibiotic suppression throughout pregnancy.

### REFERENCES

1. Gilstrap LC II, Cunningham FG, Whalley PF: Acute pyelonephritis in pregnancy: An anterospective study. Obstet Gynecol 57:409–413, 1981.
2. Lenke RR, VanDorsten JP, Schifrin BS: Pyelonephritis in pregnancy: A prospective randomized trial to prevent recurrent disease evaluating suppressive therapy with nitrofurantoin and close surveillance. Am J Obstet Gynecol 146:953–957, 1983.
3. Faro S, Pastorek JG II, Plauche WC, Korndorffer FA, Aldridge KE: Short-course parenteral antibiotic therapy for pyelonephritis in pregnancy. South Med J 77:455–457, 1984.
